# Stimulation of ribosomal frameshifting by RNA G-quadruplex structures

**DOI:** 10.1093/nar/gkt1022

**Published:** 2013-10-30

**Authors:** Chien-Hung Yu, Marie-Paule Teulade-Fichou, René C. L. Olsthoorn

**Affiliations:** ^1^Department of Molecular Genetics, Leiden Institute of Chemistry, Leiden University, PO Box 9502, Leiden, The Netherlands and ^2^Institut Curie, UMR 176-CNRS, Bât 110, Université Paris-Sud, 91405 Orsay, France

## Abstract

Guanine-rich sequences can fold into four-stranded structures of stacked guanine-tetrads, so-called G-quadruplexes (G4). These unique motifs have been extensively studied on the DNA level; however, exploration of the biological roles of G4s at the RNA level is just emerging. Here we show that G4 RNA when introduced within coding regions are capable of stimulating −1 ribosomal frameshifting (−1 FS) *in vitro* and in cultured cells. Systematic manipulation of the loop length between each G-tract revealed that the −1 FS efficiency positively correlates with G4 stability. Addition of a G4-stabilizing ligand, PhenDC3, resulted in higher −1 FS. Further, we demonstrated that the G4s can stimulate +1 FS and stop codon readthrough as well. These results suggest a potentially novel translational gene regulation mechanism mediated by G4 RNA.

## INTRODUCTION

G-quadruplex (G4) structures formed by guanine (G)-rich nucleic acid sequences are characterized by their four-stranded G-tracts in combination with multiple stacked G-quartets. As opposed to typical Watson–Crick base pair forming duplexes, G-quartets are constituted by noncanonical Hoogsteen hydrogen bonds between these G bases. Although both RNA and DNA can adopt G4s, structural analysis demonstrates that RNA G4s fold into parallel-stranded conformations independent of nucleotide sequences, the species of cations and the concentration of RNA molecules (1). DNA G4s show structural polymorphism according to various factors (2,3). These polymorphic structures have been shown to correlate with biological functions (4,5).

As opposed to extensive studies on DNA G4s, our knowledge of RNA G4s, especially their biological consequences, remains limited. Recent advances have demonstrated that RNA G4s are key players in various cellular functions, including telomere homeostasis, pre-mRNA processing (splicing and polyadenylation), mRNA targeting, RNA turnover and translation (6). Among these characterized functional roles, RNA G4s located within the 5′-untranslated regions (5′-UTRs) in relation to translational control are best studied. Several mechanisms related to translation initiation have been proposed to explain the roles of G4s in 5′-UTR: (i) interference with cap binding by the eIF4F complex (7); (ii) steric hindrance of start codon recognition (8); (iii) impeding the scanning process of ribosomal 40S subunit (9–11); (iv) assisting in formation of internal ribosomal entry site for cap-independent translation initiation (12). Interestingly, a direct correlation between thermodynamic stability of RNA G4s in 5′-UTRs and their ability to repress translation has been shown (13), suggesting that RNA G4s can act as tunable roadblocks to control gene expression by affecting ribosome scanning (14).

−1 ribosomal frameshifting (−1 FS) is a translational recoding mechanism whereby translating ribosomes are forced to move one nucleotide (nt) backward, leading to the decoding of a second open reading frame (ORF) located in the −1 register with respect to the first ORF (15–17). Two elements within mRNA are required to induce efficient −1 FS: a 7-nt slippery sequence where FS occurs (18), and a stimulatory structure that can be a pseudoknot, a hairpin or antisense oligonucleotide-forming duplex (19,20) located 5–8 nt downstream of the slip site. Several models have been proposed to explain the mechanism of −1 FS (21–23). One generally accepted feature is that the mechanical stability of the downstream structure is critical to −1 FS, but a simple correlation between stability and frameshifting efficiency is not evident (24–27).

Because stable RNA G4s in the 5′-UTRs can impede 40S ribosomal subunit scanning, and stable structures are required to stall translating ribosomes to induce −1 FS, we hypothesized that G4 RNAs in the coding region can stall ribosomes, owing to their unusual stability, and thus promote −1 FS. While ribosomal stalling by G4s has recently been demonstrated in a bacterial system (28), we demonstrate here that natural and synthetic G4 RNA motifs are indeed efficient frameshifting signals in a mammalian system.

## MATERIALS AND METHODS

### Frameshift construct and oligonucleotides

−1 FS was monitored by the pSF208 construct described earlier (29). Sets of complementary oligonucleotides (Sigma-Aldrich) were annealed, followed by ligation into SpeI and NcoI digested pSF208. To monitor +1 FS and stop codon readthrough (RT), pSF208 was digested by BglI/NcoI, followed by insertion of annealed synthetic dsDNA fragments. A list of oligonucleotides is available on request. All constructs were verified by automated dideoxy sequencing using chain terminator dyes (LGTC, Leiden).

### *In vitro* transcription and *in vitro* translation

Plasmid DNA was linearized by BamHI, followed by successive phenol/chloroform extraction and ethanol precipitation. SP6 RNA polymerase-directed transcriptions were carried out according to manufacturer's protocol (Promega). After transcription, RNA samples were loaded on a 1% agarose gel to determine the quality and quantity. Appropriate dilutions of the transcription mixtures in RNase-free water were directly used for *in vitro* translation. The translation mixtures (10 µl) contained 5 nM of mRNA, 4 µl of nuclease-treated rabbit reticulocyte lysate (RRL, Promega), 0.5 µl of 1 mM amino acids mix (Promega) without methionine, 0.25 µl of ^35^S-methionine (>1000 Ci (37.0TBq)/mmol, EasyTag, Perkin Elmer), and indicated amounts of PhenDC3 (30) or TMPpyP4 (31), and were incubated at 28°C for 1 h. Translation reactions were terminated by adding equal volume of 2× Laemmli buffer followed by heating up to 80°C for 5 min. Samples were separated by 13% of sodium dodecyl sulphate (SDS)-polyacrylamide gels. Gels were dried and then exposed to phosphoimager screens (Molecular Dynamics). Band intensity of in-frame and recoding products (including −1 FS, +1 FS and RT) were measured by Molecular Imager FX (BioRad) or Typhoon 9400 scanner (GE Healthcare), and were quantified by Quantity One software (BioRad). Frameshifting efficiency was calculated as the amount of recoded products divided by the sum of in-frame and recoded product, corrected for the number of methionines (10 in the 0-frame product and 28 in the recoded products), and multiplied by 100.

### Frameshift assays in mammalian cells

Selected G4 constructs were tested in HEK293T cells using the dual luciferases reporter, pDUAL-HIV(0), as described earlier (29). In short, pDUAL-HIV(0) was digested by KpnI/BamHI, followed by insertion of complementary oligonucleotides. HEK293T cells were cultured in Dulbecco's modified Eagle's medium/high glucose/stable glutamine (PAA Laboratories) supplemented with 10% fetal calf serum and 100 U/ml penicillin and 100 µg/ml streptomycin. Cells were kept in a humidified atmosphere containing 5% CO_2_ at 37°C on a regular subculturing regime. Cells were transfected with 300 ng of plasmid by 1 µl of Lipofectamine 2000 (Invitrogen) in a 24-well culture plate. Cells were lysed 20–24 h after transfection and luciferase activities were measured by GLOMAX multidetector (Promega) using Dual-Luciferase Reporter Assay Kit (Promega). Frameshifting efficiency was obtained by dividing the ratio Renilla luciferase (RL) over Firefly luciferase (FL) activity of the mutant by the RL/FL ratio of the in-frame control and multiplied by 100.

## RESULTS

### Endogenous RNA G4s derived from natural 5′-UTRs can induce −1 FS

**Figure 1.**
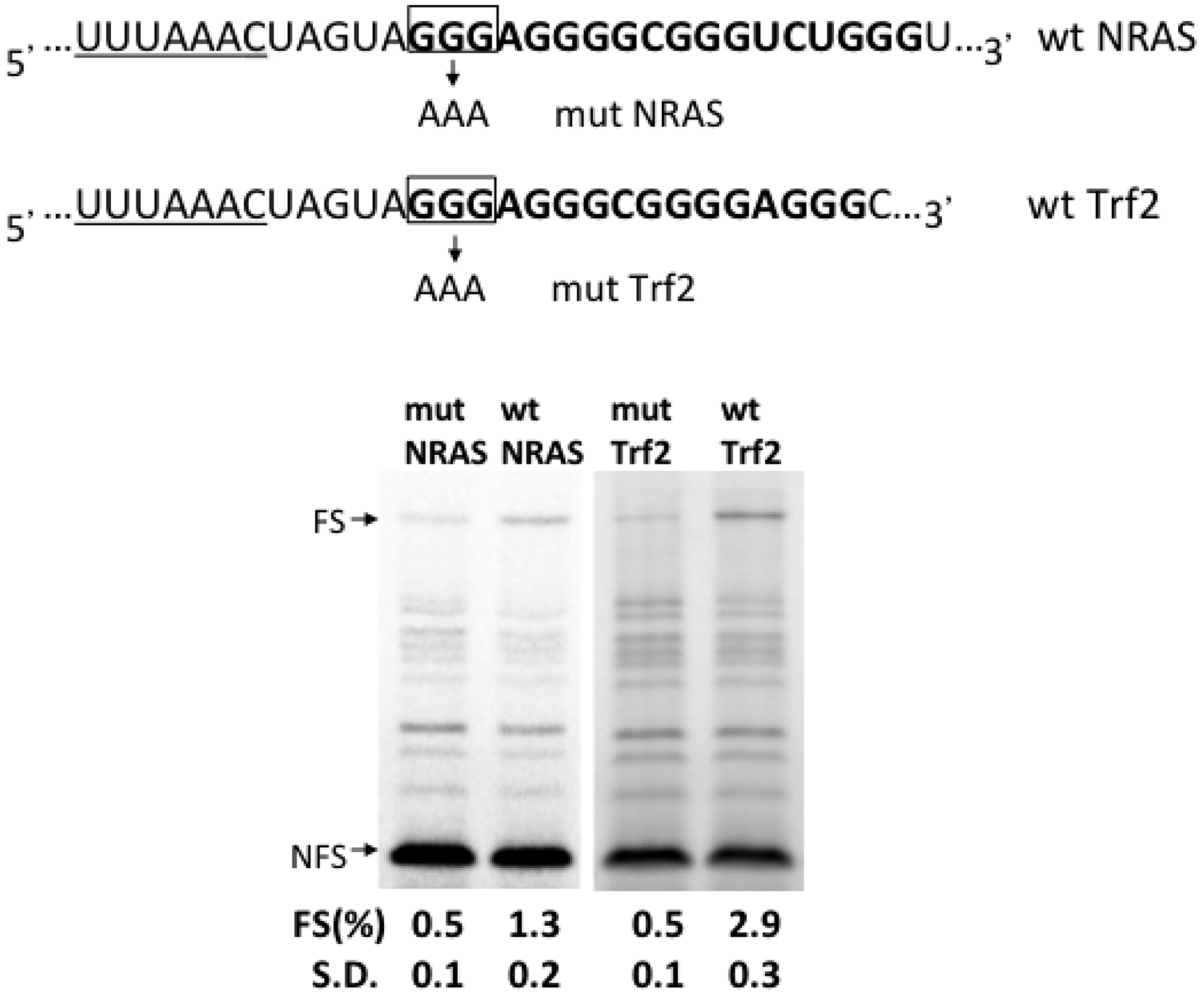
Natural RNA G4s can cause −1 frameshifting (−1 FS). The wild-type (wt) G4 forming sequences located in the 5′-UTR of NRAS (wt NRAS) and Trf2 (wt Trf2) were cloned 5-nt downstream of UUUAAAC slippery sequence (underlined) in a frameshifting reporter construct (33). Mutants, mut NRAS and mut Trf2, that are unable to form a G4 were constructed by replacing the 5′ proximal G-tract by 3 As. SDS-PAGE analysis was used to resolve the ^35^S-methionine labeled translation products of the indicated constructs in rabbit RRL. −1 FS is monitored by the presence of a 65-kD product, indicated by ‘FS’. The 0-frame product is indicated by ‘NFS’. Quantitative analysis of frameshifting efficiency [FS (%)] is described in ‘Materials and Methods’ section. The standard deviation (S.D.) is derived from at least three independent experiments.

To investigate the possibility of RNA G4 in inducing −1 FS, we first examined two well-defined suppressive RNA G4s located in the 5′-UTR of NRAS (9) and Trf2 (32) to prove our principle. As shown in Figure 1, both RNA G4s when located 5 nt downstream of an efficient U_3_A_3_C slippery sequence showed significant −1 FS compared with corresponding negative controls in an *in vitro* translation assay (2.6-fold and 6-fold for NRAS and Trf2, respectively). These data indicate that the stable RNA G4s, which can interrupt 40S ribosomal subunit scanning are able to act as roadblocks to stimulate frameshifting as well.

### Spacer length effect of G4-induced −1 FS

**Figure 2.**
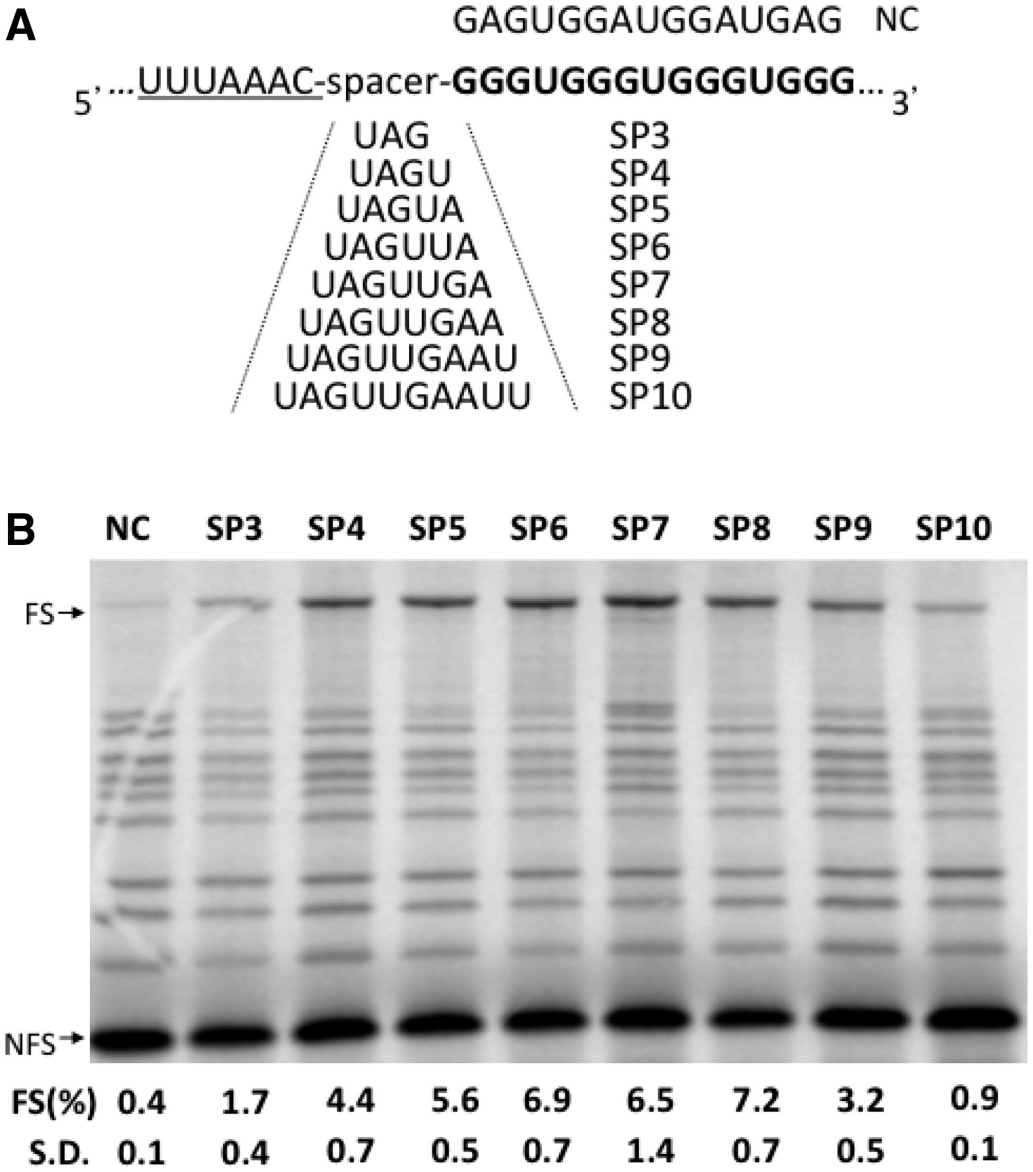
Effect of spacer-length on G4-induced −1 FS. (**A**) Sequence of different spacers between slippery sequence and G4 structures is shown. The sequence of the negative control (NC), which cannot form a G4 is also shown. (**B**) SDS-PAGE analysis of ^35^S-methionine–labeled translation products using various spacer-length (SP3-SP10) constructs. See legend to Figure 1 for details.

An optimal spacer length between slippery sequence and downstream stimulatory structures is crucial for efficient −1 FS (34,35). Although generally between 5 and 8 nt, the optimal distance depends on individual frameshifting signals. To investigate the optimal distance of RNA G4s, a novel frameshifting signal in inducing −1 FS, we increased the spacer length stepwise from 3 to 10 nt in the (G_3_U)_4_ background (Figure 2A). (G_3_U)_4_ has been reported as the most stable G4 structure both at RNA and DNA level (36,37) and thus may result in higher FS efficiency. Almost 2-fold higher FS than the Trf2 G4 was measured at the same spacer length of 5 nt (Figure 2B, SP5), while a control construct, in which the G4 structure is disrupted by 4 G-to-A mutations showed only 0.4% of FS (Figure 2B, NC). FS reached an optimum of ∼7% at a spacer length of 6–8 nt (Figure 2B, SP6-SP8).

The −1 FS efficiency was also tested in cultured mammalian cells using a dual luciferase reporter plasmid (33) The FS efficiency *in vivo* was related to an in-frame control whose activity was set at 100% (see ‘Materials and Methods’ section). Similar to *in vitro* experiments, optimal FS was observed for the 6-nt spacer with decreasing FS efficiencies for shorter and longer spacers (Supplementary Figure S1). In general, FS efficiencies measured *in vivo* are much lower than those obtained *in vitro* [see e.g. (29)].

### −1 FS efficiency is positively correlated with the thermodynamic stability of G4 RNA

**Figure 3.**
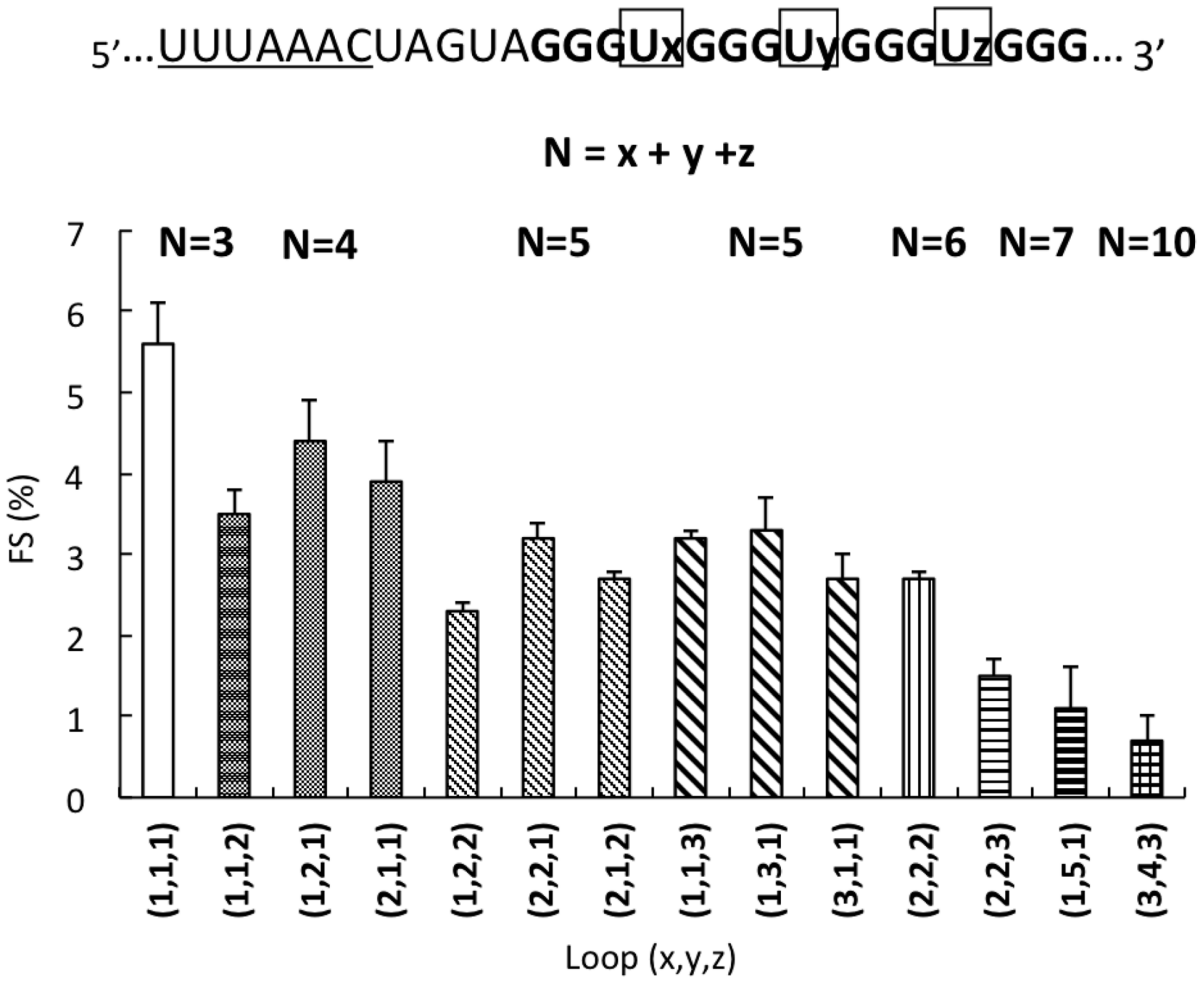
Effect of loop length of RNA G4-induced −1 FS. The G4 sequence is composed of three loops denoted as x, y and z (from 5′ to 3′). N represents the sum of nucleotides in all three loops. The graph shows the results of −1 FS (indicated by bars, y-axis) induced by G4 with various loops {including different number of nucleotides at different positions [indicated by (x,y,z)] and total numbers (N, above the graph)}.

A typical feature of G4 RNAs is that the four G3 tracts are separated by three loops. The length of these loops is central to G4 stability and topology (36). To better characterize RNA G4s as frameshifting signals, we systematically investigated the effect of total loop length as well as the orientation of the loop in −1 FS using the representative U-rich sequence in these loop regions. To clarify, the number of the first, second and third loop regions separated by four G3 tracts were denoted as (x, y, z) (Figure 3). For example, a total loop-length of 4 nt can result in three different constructs with loop orientation as (1,1,2), (1,2,1) and (2,1,1), respectively. These three loop variants induced 3.5, 4.4 and 3.9% of −1 FS, respectively (Figure 3 and Supplementary Figure S2). For the six constructs with total loop length of 5 nt, the −1 FS efficiency ranges from 2.3 to 3.2% (Figure 3 and Supplementary Figure S2). Constructs with a total loop length of 6 (2,2,2), 7 (2,2,3 and 1,5,1) and 10 (3,4,3) nt displayed a decreasing ability in promoting −1 FS. In combination with previous data showing that the thermodynamic stability of RNA G4s is inversely correlated with total loop length (37), our results suggest that RNA G4s can induce −1 FS in a thermodynamic stability-dependent manner. We and others previously observed the same trend for frameshifting signals formed by perfect stem-loop structures (29) and antisense oligonucleotides (20,21,38).

We further increased the number of G3 repeats with the aim to induce higher −1 FS due to their better ability to impede ribosomal scanning (13). However, with the exception of (G_3_U)_5_, we observed less efficient −1 FS by incrementing the number of G_3_U repeats (Supplementary Figure S3, left). These data are in contrast to a previous study applying G4 RNAs in the 5′-UTR as translational suppressors (13). This discrepancy may be due to the intrinsic differences of the readouts. To induce −1 FS, the frameshifting signal should be present at a precisely defined distance, whereas for inhibition of scanning, the precise location of the roadblock is not important. With increasing number of G3 tracts, the possibility of forming a (G_3_U)_4_ at the right distance from the slippery sequence will decrease, thus actually resulting in a decrease in FS. For obstructing ribosomal scanning, distance is not an issue, and as melting of the first G-stretch still allows formation of a new G4 by downstream G-tracts, scanning inhibition is enhanced by increasing numbers of G-tracts. Moreover, decreasing or increasing the number of G-quartets results in less FS (Supplementary Figure S3, right) in agreement with their lower thermodynamic stability (3).

### Ligands that bind G4 RNA can either enhance or decrease FS

**Figure 4.**
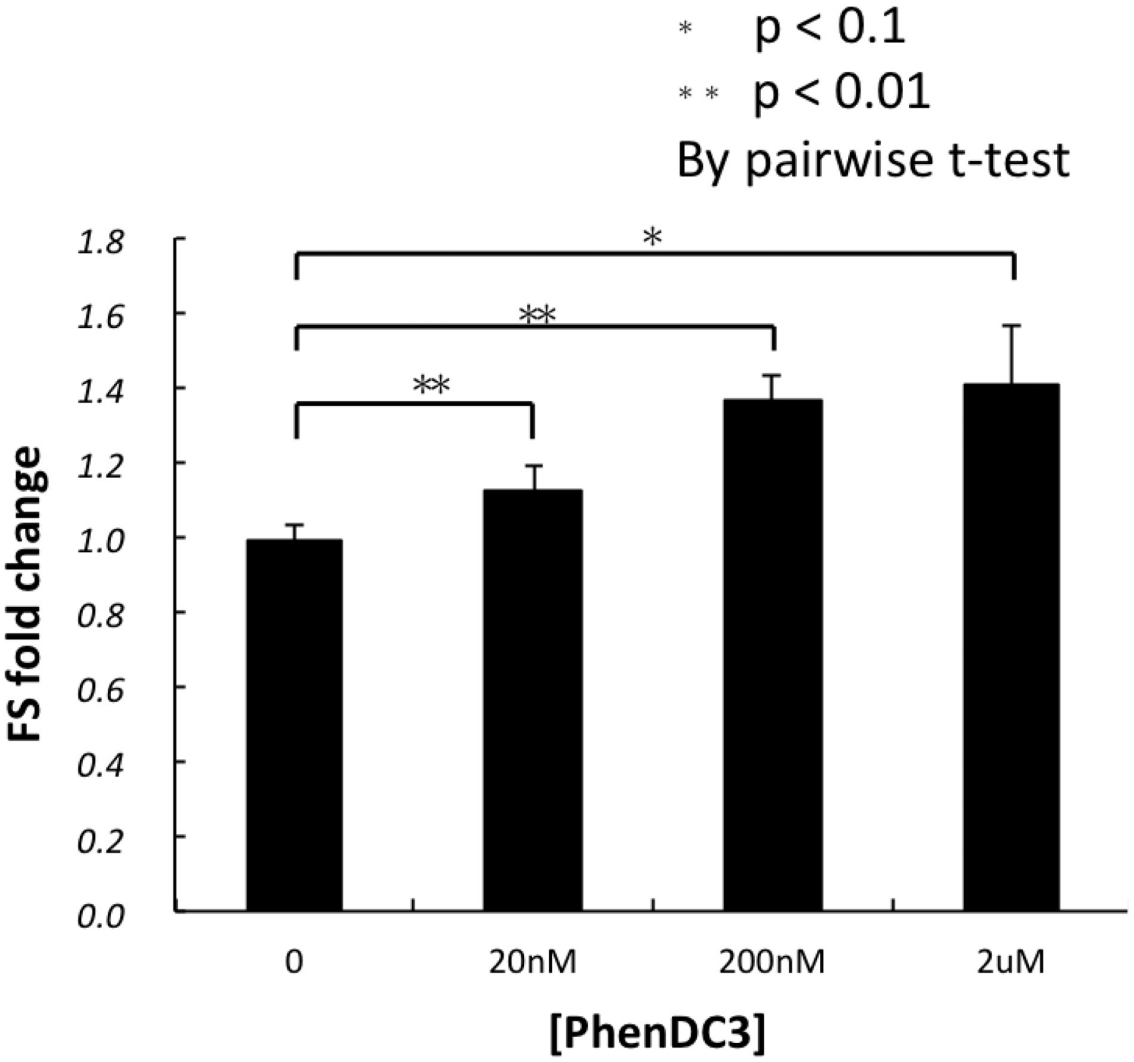
Enhancement of −1 FS by a G4-stabilizing ligand. Various concentrations (0–2 μM) of a G4-specific bisquinolinium compound, PhenDC3, were incubated with the SP5 mRNA and assayed for −1 FS efficiency in RRL. See legend to Figure 1 for more details.

Next we investigated the effect of G4 binding ligands on FS efficiency. We chose the G4 stabilizing ligand PhenDC3, which has been reported to increase the stability of a variety of DNA (30) and RNA (39) G4s and the porphyrin TMPyP4, which is a known G4 destabilizing agent (31). Addition of PhenDC3 resulted in a dose-dependent enhancement of FS of the (G_3_U)_4_ construct reaching a 1.4-fold increase at a concentration of 2 µM (Figure 4 and Supplementary Figure S4). Addition of TMPyP4 at 2 µM, although affecting global translation, decreased FS ∼3-fold (Supplementary Figure S5), while both TMPyP4 and PhenDC3 had no significant effects on frameshifting induced by a 12 base-pair hairpin (Supplementary Figure S5). These data verify that RNA quadruplex formation is responsible for the observed FS.

### G4 RNA can induce +1 FS and stop codon RT

**Figure 5.**
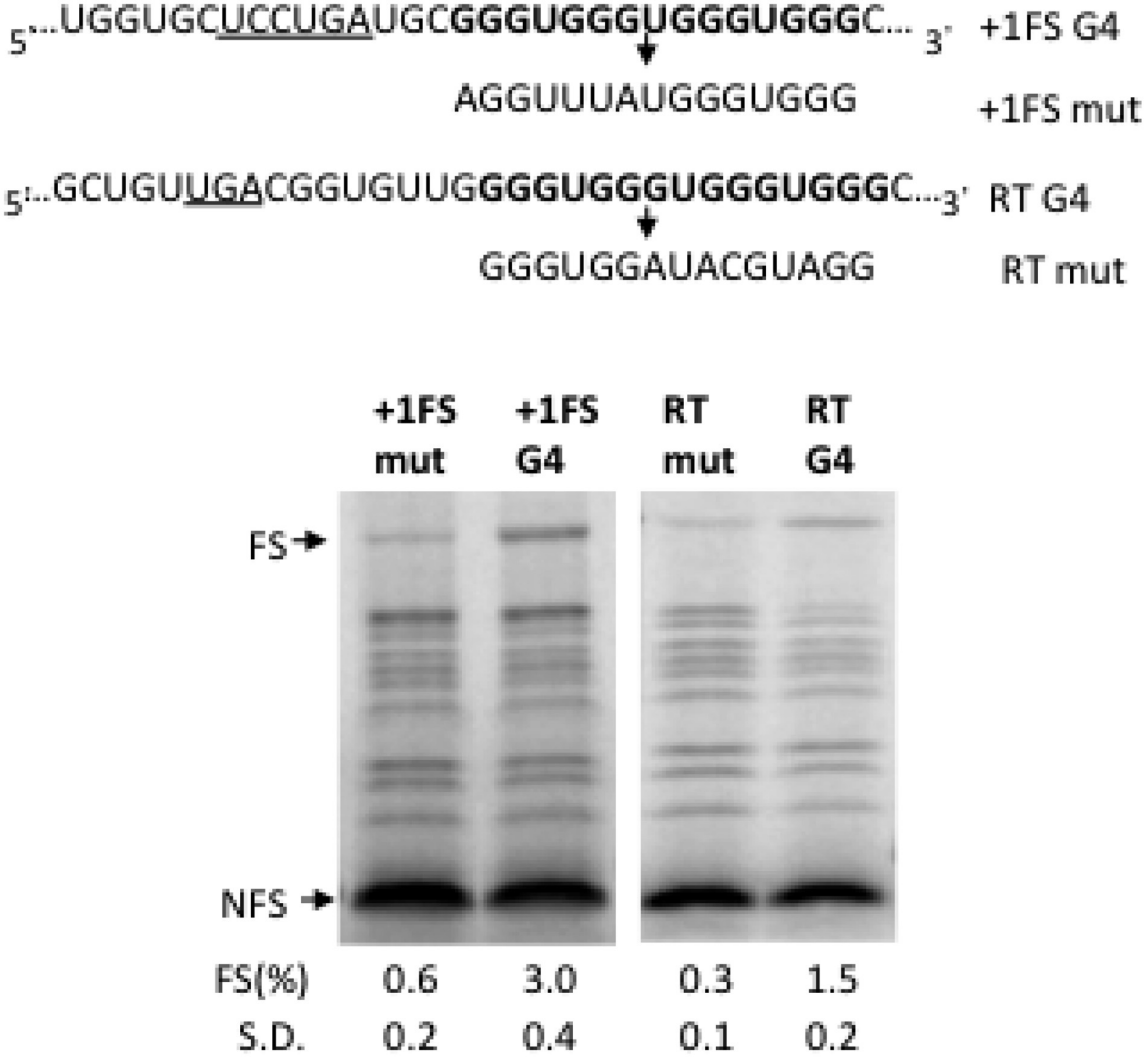
G4 can induce +1 FS and stop codon RT. The +1 FS sequence based on the antizyme gene is derived from P2lucAZ1wt (44) except for the frameshifting pseudoknot, which was replaced by (G3U)4 (‘+1FS G4’). The sequence of corresponding negative control (‘+1FSmut’) is indicated. The slip site is underlined. The stop codon RT construct is based on the Colorado tick fever virus segment 9 RT construct (43) except for the RT signal, which was replaced by (G3U)4 (‘+1FS RT’). The RT stop codon is underlined and the sequence of corresponding negative control (‘RTmut’) is indicated. An SDS-PAGE analysis of ^35^S-methionine–labeled translation products obtained by the indicated constructs in RRL is shown. See legend to Figure 1 for details.

Although the mechanism of inducing −1 FS is distinct from +1 FS or stop codon RT, a common feature of these recoding events is the involvement of a 3′ stimulatory RNA structure (40,41). To investigate if RNA G4 can induce +1 FS or stop codon RT, we replaced the well-characterized +1 FS stimulatory pseudoknot of mammalian antizyme (42) and the RT stimulatory stem-loop structure of Colorado tick fever virus segment 9 (43) with the most stable (G_3_U)_4_ G4 sequence (Figure 5). Interestingly, the RNA G4 could induce significant levels of +1 FS (3.0%) and stop codon RT (1.5%) against a background of 0.6 and 0.3%, respectively (Figure 5).

## DISCUSSION

In our present study, we demonstrated that RNA G4s can act as translational recoding signals to induce both −1 and +1 FS as well as stop codon RT. This suggests a potentially novel translational gene regulation mechanism mediated by RNA G4s. Given the high number of potential G4 forming sequences in the human genome (45), it is likely that some are present within coding regions and are involved in translational recoding. Recently, some G4s present in bacterial mRNAs were reported to induce ribosomal stalling (28). Although the function of this stalling remains unknown, ribosomal frameshifting was suggested to be one of the possibilities.

The highest level of −1 FS that we achieved with a G4 RNA is 7%, using (G_3_U)_4_ as stimulator. Although this level is rather modest compared with levels obtained with pseudoknot-stimulated FS, which can reach >40% as in the case of the ‘Infectious Bronchitis virus’ FS pseudoknot (46), it is significantly higher than some natural FS signals like those present in Influenza A virus PA gene whose FS efficiency is 2% (47)_._ A FS efficiency of 7% is comparable with that obtained with a hairpin of 8 bp with a calculated Δ*G* of −17.1 kcal/mol (29). Interestingly, the stability of (G_3_U)_4_ has been measured to be only −8.16 kcal/mol (37). So, how is this small structure of 15 nt capable of redirecting 7% of ribosomes into another reading frame? The answer lies probably in the peculiar topology of the G4 structure comprising eight hydrogen bonds and four purine stacks per helical step, making it difficult for the ribosome to melt the first G-quartets that reside at the opening of the mRNA entrance tunnel. The stability of the first 3 or 4 bp has also been shown to be critical for shifting the ribosome by hairpins (29,48) and antisense LNA oligonucleotides (38).

The (G_3_U)_4_ sequence was also capable of stimulating +1 FS as well as stop codon RT with an efficiency of 3.0 and 1.5%, respectively. Although these values may seem low, *in vitro* RT frequencies reported for CTFV are between 3.6 and 6.7% (43) and for Alphaviruses are 6.4–7.6% against a background of 0.8–2.0% in the absence of a stimulatory structure (49). The +1 FS efficiency of the antizyme pseudoknot in the absence of spermidine is 2–3% (42), which is comparable with our G4-stimulated +1 FS efficiency. A possible stimulating effect of spermidine on G4-mediated +1 FS was not investigated by us.

We have also investigated the effect on −1 FS of several known G4 ligands. The bisquinolinium derivative PhenDC3, which is a general stabilizer of RNA G4s (39), was found to enhance −1 FS efficiency ∼1.5-fold, whereas TMPyP4, known to destabilize certain RNA G4s (31), reduced FS ∼3-fold. Other DNA G4 stabilizing ligands like 2,4-Bis-[(E)-4-(dimethylaminostyryl)]-1-[4-(triethylammonio)butyl]pyridinium dibromide [Distyryl 1b (50)] and 4a,10a,16a-triazoniatrinaphthylene [TrisQ (51)] had no effect on −1 FS (data not shown). These ligands though have strong DNA sequence and/or structure preferences and may not stabilize (G_3_U)_4_ RNA.

One of the interesting aspects of G4s is their stabilization by potassium ions (52,53). This would allow a potential natural G4-dependent FS signal to be able to respond to changing cellular or environmental conditions. We have not investigated this possibility here since the lysate used in our *in vitro* translation assays already contains a high level of potassium (57 mM, Promega Technical Manual 232) but it is conceivable that at even higher concentrations of K^+^ (>100 mM), FS may be enhanced. In addition to potassium, several proteins like FMRP (54) and RHAU or DHX36 (55) are known to bind RNA G4s and could play a regulatory role in this type of FS.

Previously, a G-rich sequence was reported to be involved in a +1 frameshift of herpes simplex virus (HSV) (56). However, in the case of HSV, the ribosomal slippage was thought to occur within the G-rich sequence itself at a frequency of ∼1%, and is not stimulated by a downstream structure, and is therefore different from our G4-stimulated FS.

In conclusion, RNA G4s are capable of stimulating −1 and +1 FS as well as stop codon RT, thereby expanding the repertoire of RNA structures involved in translational recoding. Whether RNA G4s are present at natural recoding sites remains to be investigated.

## SUPPLEMENTARY DATA

Supplementary Data are available at NAR Online.

## FUNDING

Funding for open access charge: Leiden Institute of Chemistry, Leiden University, Leiden, The Netherlands

*Conflict of interest statement*. None declared.

## Supplementary Material

Supplementary Data
