# Supplementary material for: Stimulation of ribosomal frameshifting by RNA G-quadruplex structures
Source: Nucleic Acids Res. 2013 Oct 30;42(3):1887–92. doi: 10.1093/nar/gkt1022 (PMC3919603; doi:10.1093/nar/gkt1022)
Supplement: Supplementary Data [file supp_42_3_1887__index.html]

Stimulation of ribosomal frameshifting by RNA G-quadruplex structures — Stimulation of ribosomal frameshifting by RNA G-quadruplex structures — Supplementary Data 

# Stimulation of ribosomal frameshifting by RNA G-quadruplex structures

## Supplementary Data

files

**Files in this Data Supplement:**

- Supplementary Data - pdf file
