# Supplementary material for: Stimulation of ribosomal frameshifting by RNA G-quadruplex structures
Source: Nucleic Acids Res. 2013 Oct 30;42(3):1887–92. doi: 10.1093/nar/gkt1022 (PMC3919603; doi:10.1093/nar/gkt1022)
Supplement: Supplementary Data [file supp_gkt1022_nar-02074-f-2013-File002.pdf]

Supporting Information
for

Stimulation of ribosomal frameshifting by RNA G-quadruplex structures

*Chien-Hung Yu[1], Marie-Paule Teulade-Fichou[2], and René C.L. Olsthoorn[1]**

[1]Department of Molecular Genetics, Leiden Institute of Chemistry, Leiden University, PO Box 9502, Leiden, The Netherlands.

[2]Institut Curie, UMR 176-CNRS, Bât 110, Université Paris-Sud, 91405 Orsay, France.

*To whom correspondence may be addressed. E-mail: olsthoor@chem.leidenuniv.nl

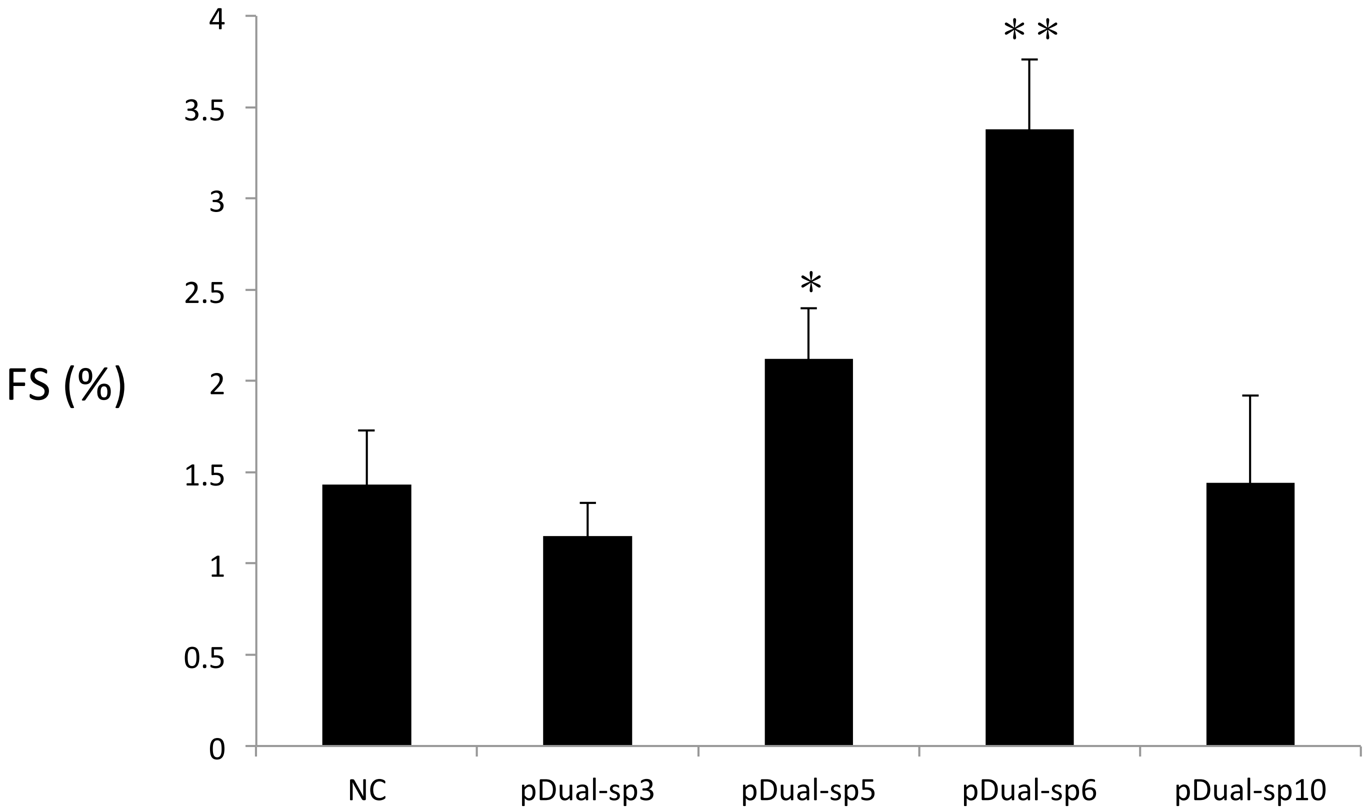

Figure S1. –1 FS in HEK293T cells. Selected spacer-length constructs were assayed for the ability to induce –1 FS in HEK293T cells. The –1 FS efficiency was obtained by measuring the Renilla and Firefly luciferases activity of the frameshift reporter constructs (see Materials and Methods). NC is a negative control in which the $(GGGU)_3GGG$ motif of pDual-sp5 was changed to AGGUGGAUGGGUGGG. The indicated efficiency and error are from at least two independent triplicate experiments. *compared to NC, $p < 0.1$; **compared to NC, $p < 0.01$.

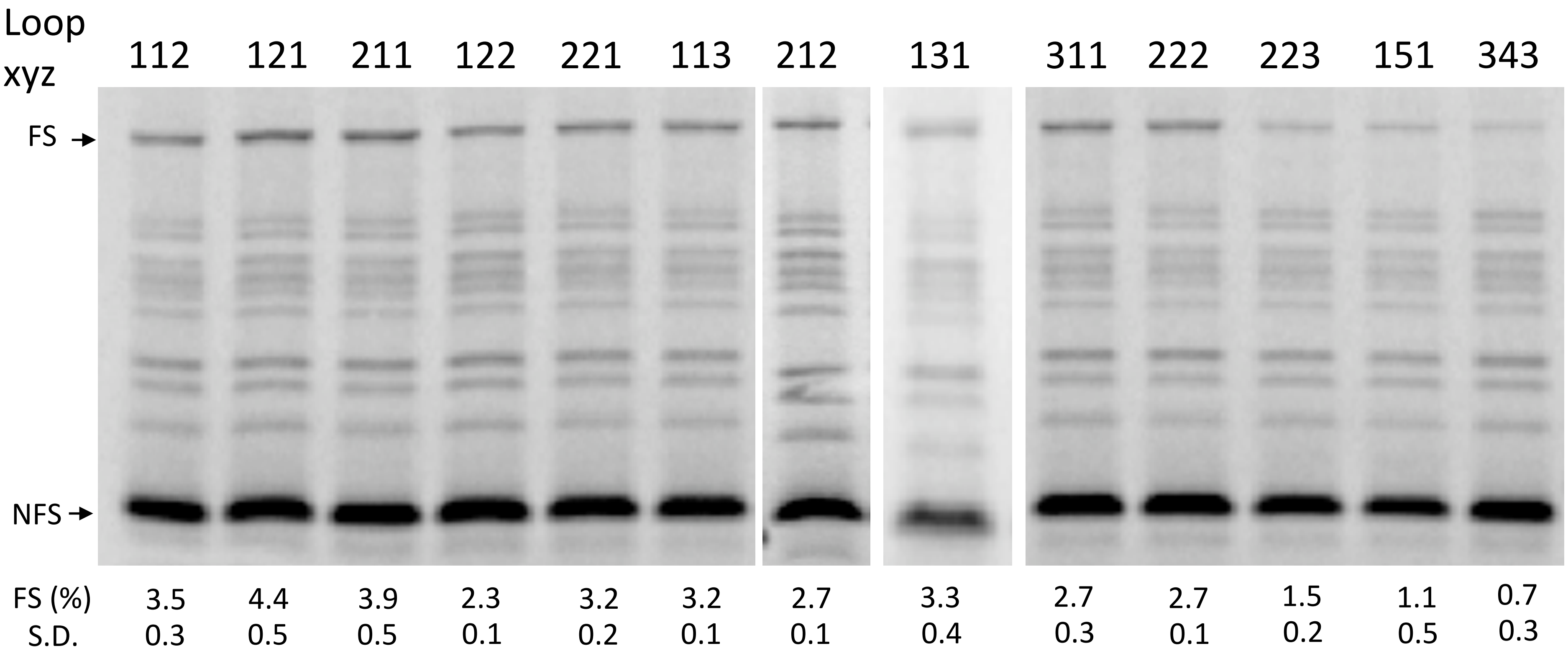

Figure S2. Effect of loop length of RNA G4 induced -–1 FS. SDS-PAGE analysis of ^{35}S-methionine labeled translation products using G4s with different loop sizes. The numbering above each lane indicates the number of U's in each of the three loops xyz in GGGUxGGGUyGGGUzGGG, with the exception of "343" where the loops are UUA, UUUU, UUA. "FS" and "NFS" indicate the frameshifted and non-frameshifted products.

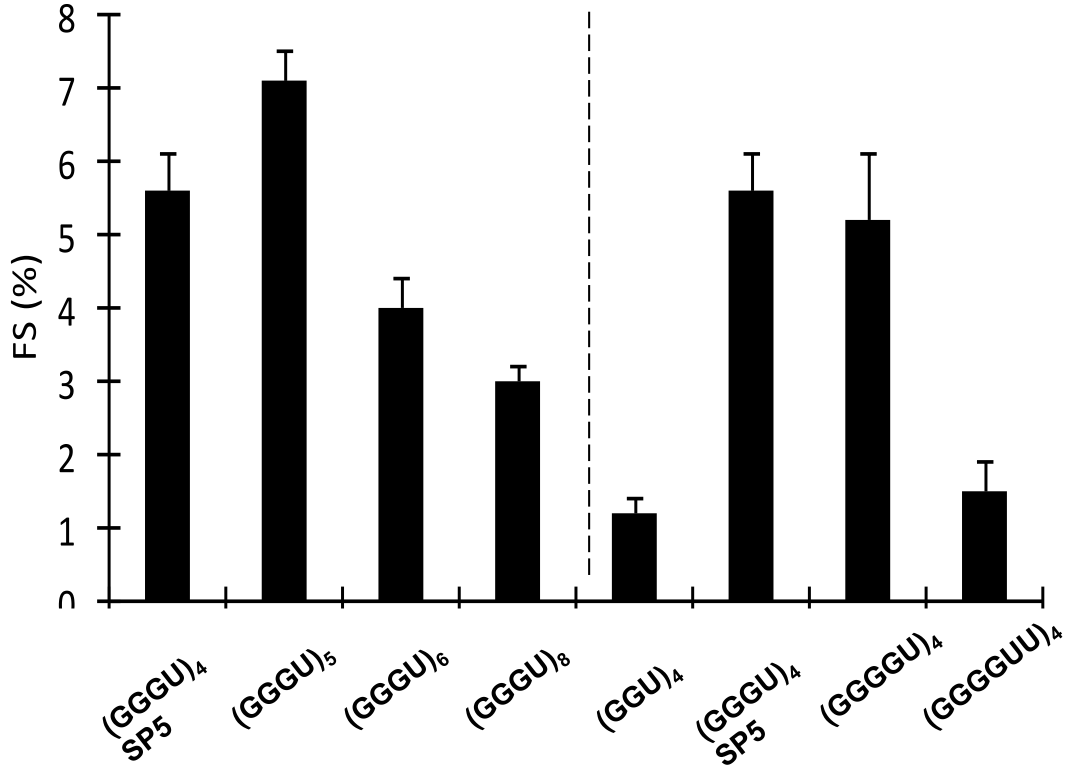

Figure S3. The –1 FS efficiency of various numbers of (G_3U) repeats (left) and various numbers of G-quartets (right). The graph shows the –1 FS efficiency (indicated by bars, y-axis) in correlation with corresponding constructs (indicated on the x-axis).

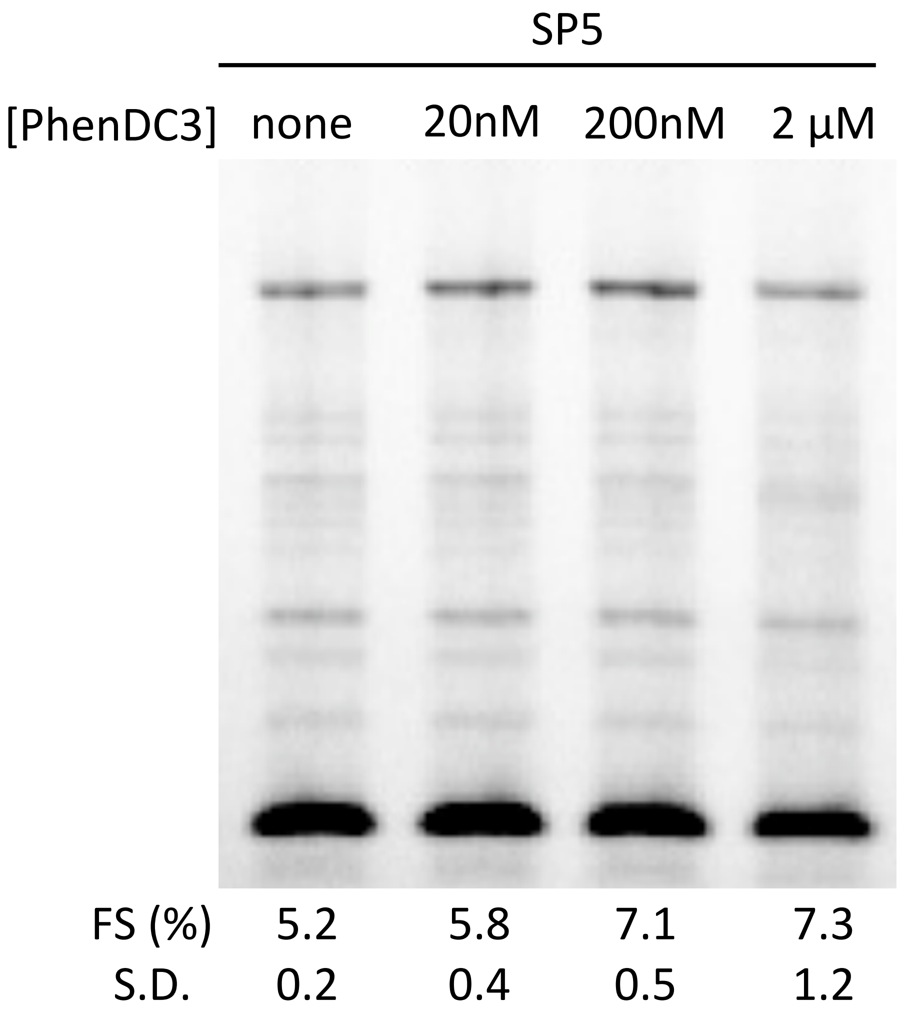

Figure S4. Enhancement of –1 FS by a G4 stabilizing ligand. Various concentrations (0-2 µM) of a G4-specific bisquinolinium compound, PhenDC3, were incubated with the SP5 mRNA and assayed for –1 FS efficiency in RRL.

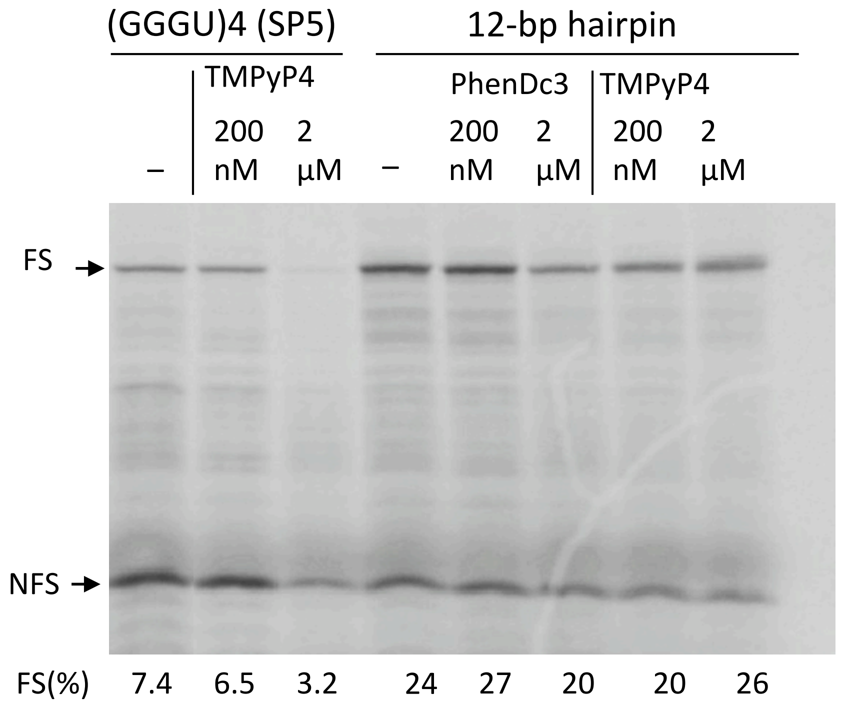

Figure S5. G4 specific ligands influence frameshifting induced by a G4 structure but not by an RNA hairpin. SDS-PAGE showing ^{35}S-methionine labeled translation products obtained by the indicated constructs in RRL in the presence of various concentrations of PhenDC3 or TMPyP4. –1 FS is monitored by the presence of a 65-kD product, indicated by “FS”. The 0-frame product is indicated by “NFS”.
